# Effects of Sevoflurane Inhalation Anesthesia on the Intestinal Microbiome in Mice

**DOI:** 10.3389/fcimb.2021.633527

**Published:** 2021-03-18

**Authors:** Ci Han, Zhaodi Zhang, Nana Guo, Xueting Li, Mengyuan Yang, Yahui Peng, Xiaohui Ma, Kaijiang Yu, Changsong Wang

**Affiliations:** ^1^Department of Critical Care Medicine, The First Affiliated Hospital of Harbin Medical University, Harbin, China; ^2^Department of Anesthesiology, Harbin Medical University Cancer Hospital, Harbin, China; ^3^Department of Critical Care Medicine, Harbin Medical University Cancer Hospital, Harbin, China

**Keywords:** inhalation anesthesia, intestinal microbiome, sevoflurane, 16S rRNA, gene sequencing

## Abstract

In recent years, more and more attention has been paid to intestinal microbiome. Almost all operations will go through the anesthesia process, but it is not clear whether the intervention of anesthesia alone will affect the change in the intestinal microbiome. The purpose of this study was to verify the effect of sevoflurane inhalation anesthesia on the intestinal microbiome. The animal in the experimental group was used to provide sevoflurane inhalation anesthesia for 4 hours. The control group was not intervened. The feces of the experimental group and the control group were collected on the 1st, 3rd, 7th and 14th days after anesthesia. Sevoflurane inhalation anesthesia will cause changes in the intestinal microbiome of mice. It appears on the 1st day after anesthesia and is most obvious on the 7th day. The specific manifestation is that the abundance of microbiome and the diversity of the microbiome is reduced. At the same time, Untargeted metabonomics showed that compared with the control group, the experimental group had more increased metabolites related to the different microbiome, among which 5-methylthioadenosine was related to the central nervous system. Subsequently, the intestinal microbiome diversity of mice showed a trend of recovery on the 14th day. At the genus level, the fecal samples obtained on the 14th day after anesthesia exhibited significantly increased abundances of Bacteroides, Alloprevotella, and Akkermansia and significantly decreased abundances of Lactobacillus compared with the samples obtained on the 1st day after anesthesia. However, the abundance of differential bacteria did not recover with the changing trend of diversity. Therefore, we believe that sevoflurane inhalation anesthesia is associated with changes in the internal microbiome and metabolites, and this change may be completed through the brain-gut axis, while sevoflurane inhalation anesthesia may change the intestinal microbiome for as long as 14 days or longer.

## Introduction

The human intestinal microbiome comprises a large number of bacteria and is the main source of intestinal bioactive substances and microbial metabolites. Due to genetic, geographical and other congenital individual differences, host individuals exhibit different intestinal microbial compositions (Roux et al., 2009). In addition, different intestinal microbiomes can also affect the host’s metabolism and immune function (Sender et al., 2016). Under normal circumstances, the gut microbiome of healthy individuals is relatively stable (Lozupone et al., 2012). In response to exposure to stress related to surgery, the human intestinal microbiome might exhibit changes in species, abundance, and metabolites (Guyton and Alverdy, 2017).

With respect to gastrointestinal surgery, stool samples from six gastric cancer patients before and after surgery were subjected to 16S rRNA gene sequencing to study the changes in the stool microbiota of these patients induced by radical distal gastrectomy (RDG). Significant changes in the composition of the intestinal microbiome, particularly the abundances of Akkermansia, Lactobacillus, and Dialister, were observed after surgery compared with the baseline (Liang et al., 2019). These changes in the intestinal microbiome after surgery have also been verified in animals. The effect of small bowel resection (SBR) on the intestinal microbiome of mice was investigated by 16S rRNA sequencing, and the diversity and composition of the intestinal contents of mice at 7 and 90 days after SBR were compared. The results revealed that SBR can cause long-term changes in the intestinal microbiome of mice that can last up to 90 days after surgery (Sommovilla et al., 2015). The results of the study indicated that the intestinal microbiome of the body significantly changes after digestive tract surgery.

In addition to digestive tract surgery, nondigestive tract surgery can also cause changes in the intestinal microbiome. Shuai Zheng et al. tested the postoperative changes in the intestinal microbiome of 40 patients with thoracic aortic dissection with abdominal complications and speculates that these changes might be related to postoperative abdominal complications (Zheng et al., 2017). Clinical data show that despite many advances in surgical techniques, the incidence of complications, such as anastomotic leakage, has not been improved (Paun et al., 2010; Shogan et al., 2013). The above-described change in the intestinal microbiome after surgery might be related to serious complications, such as postoperative infections and anastomotic fistulas (Lederer et al., 2017; Gaines et al., 2018; Reddy et al., 2018; Gershuni and Friedman, 2019).

The impact of surgery on the intestinal microbiome might currently include (Roux et al., 2009) the application of antibiotics (Sender et al., 2016), intestinal preparation (Lozupone et al., 2012), nutrition, and (Guyton and Alverdy, 2017) surgical methods (Lederer et al., 2017). However, almost all operations will involve the use of anesthesia, but whether anesthesia alone affects the change in the intestinal microbiome remains unclear. Few studies have investigated the effects of anesthesia on the intestinal microbiome. The purpose of this study was to verify the effect of sevoflurane inhalation anesthesia on the intestinal microbiome. We used 16S rRNA gene sequencing to monitor the dynamic changes in the intestinal microbiome of the experimental and blank control groups on the 1st, 3rd, 7th and 14th days after sevoflurane inhalation anesthesia. Subsequently, Untargeted metabonomics was performed on the 7th feces of the experimental group and the control group.

## Materials and Methods

### Ethical Statement

The authors are accountable for all aspects of the work in ensuring that questions related to the accuracy or integrity of any part of the work are appropriately investigated and resolved. Experiments were performed under a project license (NO.: KY2018-02) granted by regional ethics board of Harbin Medical University Cancer Hospital on December 2018, in compliance with Chinese guidelines for the care and use of animals. The chairperson of the ethics committee is Changhong Zhao.

### Animals

SPF-grade, 6-8-week-old, Institute of Cancer Research (ICR) mice were provided by Beijing Weitonglihua Experimental Animal Co., Ltd. The mice were placed in a standard squirrel cage under controlled laboratory conditions (22°C and 12-hour light/12-hour dark cycle) and given free food and water. The mice were randomly divided into an experimental group and a control group, and the two groups were housed separately in different cages, and the mouse tails were labeled with numbers. Two mice from each group were placed in the same cage and fed.

### Experimental Design

SPF-grade, 6-8-week-old ICR, male mice were selected, and 16 were randomly divided into two groups of eight mice each. The mice in the experimental group were placed into the anesthesia box, and the animal anesthesia machine was used to provide sevoflurane inhalation anesthesia for 4 hours. The temperature of the experimental environment was controlled at 28 °C to ensure the low temperature of mice. The monitor was used to continuously monitor the concentrations of sevoflurane and oxygen in the anesthesia box and deliver sevoflurane at a rate of 5 L/min. The gas flow was adjusted to maintain a stable MAC (minimum alveolar concentration) value of 1.3, which was equivalent to 2.21% sevoflurane. The gas we used was fresh air, we monitored the oxygen concentration to be 20%, which is consistent with the oxygen content of fresh air. The spontaneous breathing of the animals was continually observed. The mice in the control group were placed in an anesthesia induction box under the same conditions but were not given sevoflurane. After the anesthesia administration period, the mice were fully awakened and returned to their original feeding environment, and their righting reflex was used as an indicator of their recovery. The feces of the experimental and control groups were collected on the 1st, 3rd, 7th and 14th days after anesthesia. The fecal collection was performed at 10 a.m. on the above-mentioned four days, and the samples were collected using sterile EP tubes and stored in a refrigerator at -80°C. We didn’t detect blood gas analysis in the experiment.

### Sample Collection

Sterile gauze was placed on the operating platform sterilized in the laboratory, and the mouse feces were collected with sterile gauze. A sterile cotton swab was used on the middle part of the mouse feces to reduce the effects of the external environment on the samples. The mouse feces were immediately placed on ice for repacking and marking. A sterile frozen storage tube was used to repack the three tubes (1-2 g in each tube), and immediately after repacking, the tubes were placed into a refrigerator at -80°C.

### Microbial Community Analysis

Microbial DNA from the fecal samples collected from the ICR mice was extracted using a DNA extraction kit according to the manufacturer’s recommended protocols (Axygen Biosciences, Union City, CA, U.S.). Primers 341f 5 ‘- cctacgggrsgcag-3’ and 806r 5 ‘- ggactacvvvgggtatctatc-3’ (with specific barcode in the primer) were used, The V3-V4 region of the bacterial 16S ribosomal RNA genes was amplified by PCR (95°C for 3 min followed by 30 cycles of 98°C for 20 s, 58°C for 15 s, and 72°C for 20 s and a final extension at 72°C for 5 min) using the primers. The PCRs were performed in a 30-µL mixture containing 15 µL of 2× KAPA Library Amplification ReadyMix, 1 µL of each primer (10 µM), 50 ng of template DNA and ddH20. Amplicons were extracted from 2% agarose gels, purified using the AxyPrep DNA Gel Extraction Kit (Axygen Biosciences, Union City, CA, USA) according to the manufacturer’s instructions and quantified using Qubit 2.0 (lnvitrogen, USA). After preparation of the library, these tags were sequenced using a MiSeq platform (Illumina, Inc., CA, USA). DNA extraction, library construction and sequencing were conducted at Realbio Genomics Institute (Shanghai, China).

### Prediction of Microbial Metabolic Function

The full name of PICRUSt (Phylogenetic Investigation of Communities by Reconstruction of Unobserved States). It infers the gene function spectrum of their common ancestors based on the gene information of OTU in greenene database. At the same time, it infers the gene function spectrum of other undetermined species in the database, constructs the gene function prediction spectrum of the whole spectrum of Archaea and bacterial domain, and finally maps the microbiome composition obtained by sequencing into the database to predict the metabolic function of the microbiome.

### Statistical Analyses

The data are presented as the means ± SEMs, and Statistical Package for the Social Sciences (SPSS v.20.0) was used for the statistical analyses. Two-way repeated-measures ANOVA was used to analyze the water maze escape latency and average speed. One-way ANOVA or unpaired t-test was used to analyze the probe quadrant trial data, the probe test data and the relative abundances of bacterial DNA. The differences were considered to be significant if p < 0.05.

## Results

**Figure 1:**
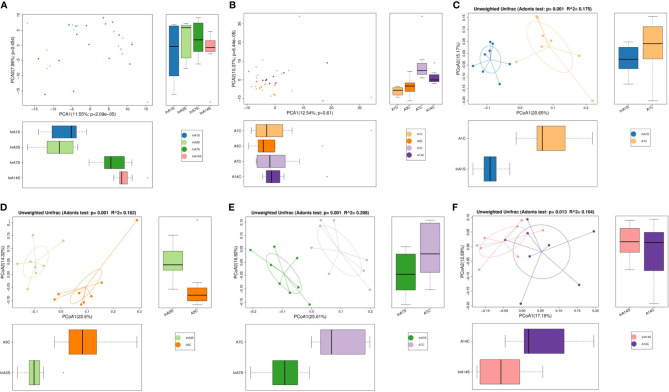
The OTU PCA and PCoA non-weighted analysis of the difference of intestinal microbiome. **(A)** The experimental group showed that the microbiome was dispersed from point to point without aggregation. **(B)** The control group was different, and the results showed the point and point aggregation (different colors in figure A and B represent different groups of samples, and the farther the distance between the two points, the greater the difference in microbial community between the two samples). **(C)** On the 1st day of the experimental group and the control group were significantly separated. **(D)** On the 3rd day of the experimental group and the control group were significantly separated. **(E)** On the 7^th^ day of the experimental group and the control group were significantly separated. **(F)** On the 14th day of the distance between the experimental group and the control group was relatively close (The points in **(C–F)** graphs represent each sample respectively, and different colors represent different sample groups).

Effect of sevoflurane inhalation anesthesia on the abundance of species in the intestinal microbiome: A principal component analysis (PCA) showed that the intestinal microbiome of the experimental mice on the 1st, 3rd, 7th and 14th days after sevoflurane anesthesia was scattered and not aggregated (**Figure 1A**).There was no significant change of intestinal microbiome in the control group (**Figure 1B**). An unweighted principal coordinate analysis (PCoA) based on the UniFrac algorithm showed significant differences between the experimental and control groups on the 1st, 3rd and 7th days after anesthesia (P = 0.001) (**Figures 1C–E**); moreover, on the 14th day, significant differences were found between the experimental and control groups, but the degree of difference had decreased compared with that detected at previous time points (P = 0.013) (**Figure 1F**).

**Figure 2:**
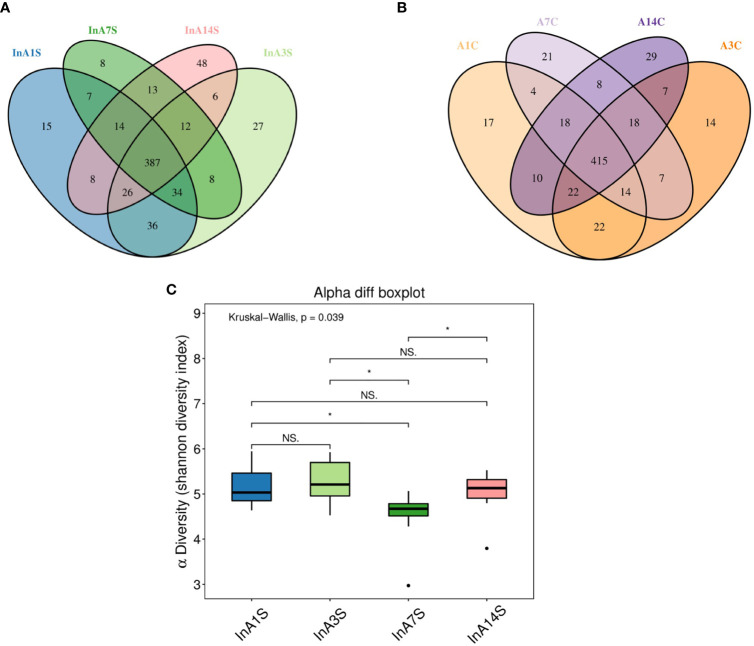
The change in intestinal microbiome abundance. **(A)** OTU Venn showed the difference in the number of OTUs in the experimental group. The number of unique OTUs on the 7th day was significantly lower than that on the 1st, 3rd and 14th day. **(B)** OTU Venn showed that there was no significant difference in the specific OTU of each day in the control group. Different color graphics represent different samples (groups), the number of overlapping parts between different color graphics is the number of OTUs shared by samples (groups); the number of overlapping parts between multiple color graphics is the number of OTUs shared by multiple samples (groups). **(C)** Analysis of the difference between groups of diversity indexes. The 7th day was significantly different from the 1st, 3rd and 14th day (the abscissa represents the sample group, and the ordinate represents the alpha diversity index value under different groups. The abnormal value is marked with “O”. When 0.01 < p < 0.05, the difference is significant, as indicated by “*”; when p < 0.01, it is extremely significant, it is indicated by “*”; “ns.” it is not significant.

The effect of sevoflurane inhalation anesthesia on the abundance of species in the intestinal microbiome was most significant on the 7th day after anesthesia. A Venn diagram of the operational taxonomic units (OTUs) showed that the experimental group had the lowest number of unique OTUs on the 7th day (**Figure 2A**), but this difference was not found in the control group (**Figure 2B**). The alpha diversity of the microbial community indicated by the Shannon index was significantly different 7 days after birth, and a trend toward recovery is detected at later time points (**Figure 2C**).

**Figure 3:**
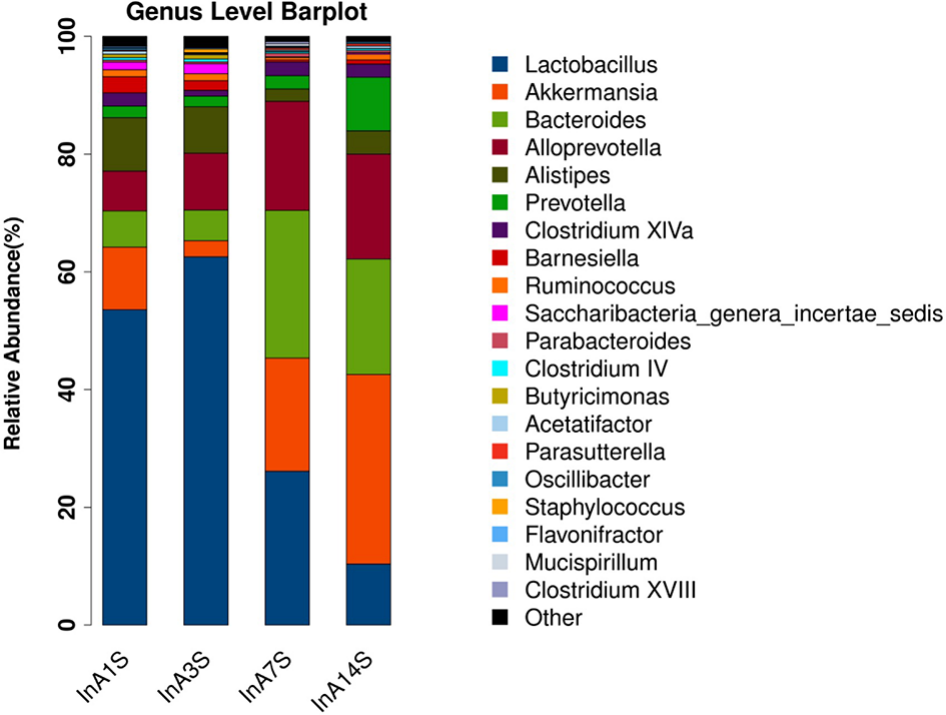
Genus difference of intestinal microbiome. Species richness analysis showed that the longer the color block, the higher the species richness. Compared with the 14th day, the color block of Bacteroides, Alloprevotella and Akkermansia increased, while the color block of Lactobacillus decreased. The abscissa is the group, and the ordinate is the relative abundance of species.

**Figure 4:**
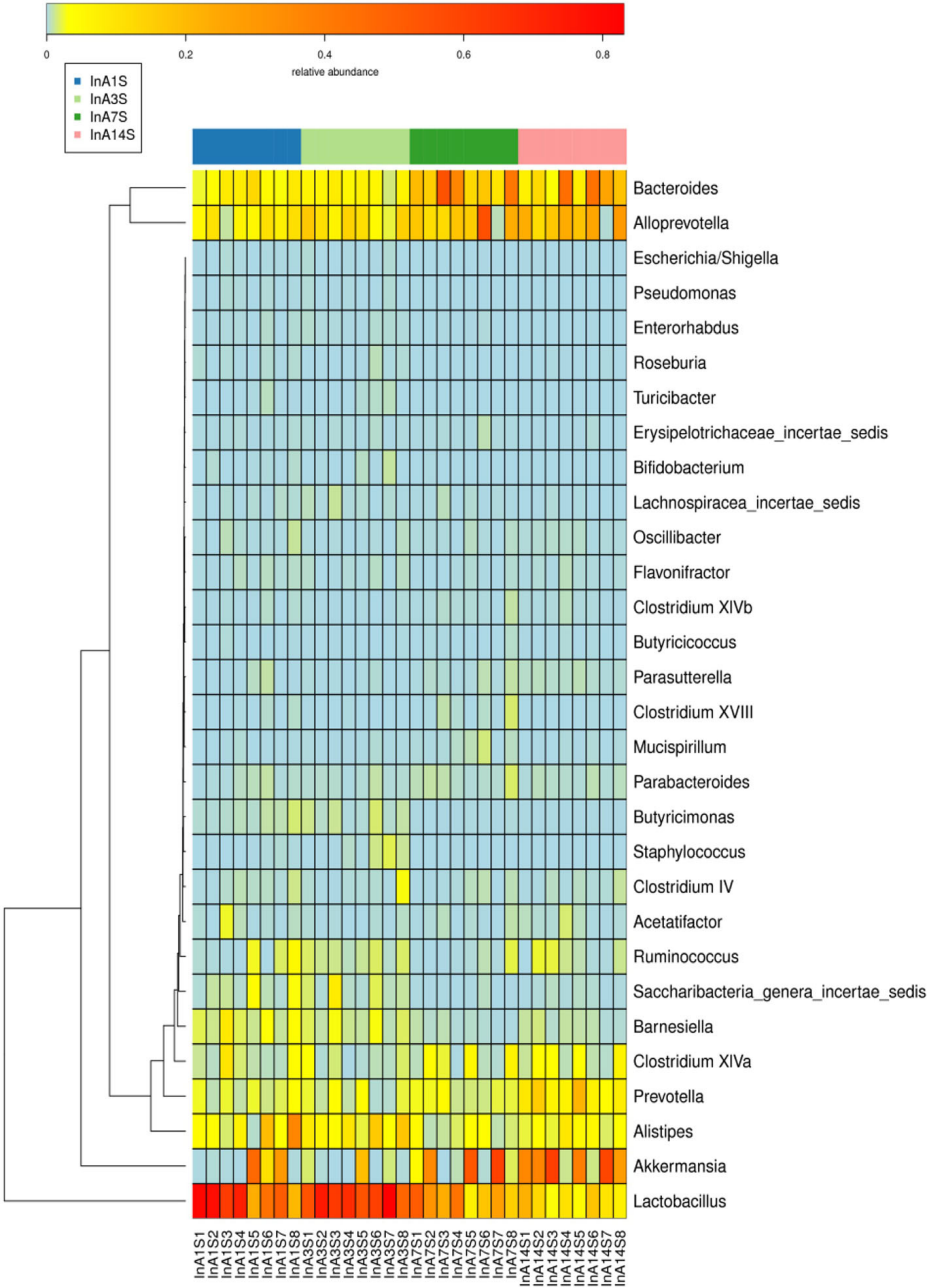
The analysis of species thermogram showed that the colors of Bacteroides, Alloprevotella and Akkermansia gradually deepened over time, while the colors of Lactobacillilus gradually became lighter over time.

Effect of sevoflurane inhalation anesthesia on change in the species comprising the intestinal microbiome in mice: At the genus level, the fecal samples obtained on the 14th day after anesthesia exhibited significantly increased abundances of Bacteroides, Alloprevotella, and Akkermansia and significantly decreased abundances of Lactobacillus compared with the samples obtained on the 1st day after anesthesia. However, the changes in Bacteroides, Alloprevotella, Akkermansia, and Lactobacillus did not recover on the 14th day (**Figures 3** and **4**).

**Figure 5:**
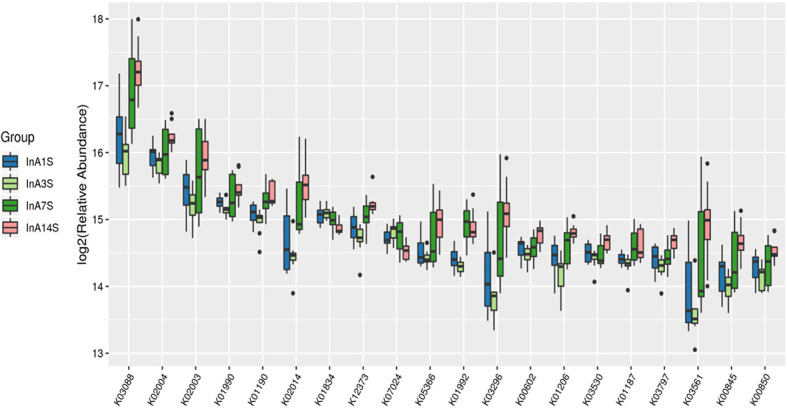
Differences in intestinal microbiome genes. KO level differential gene analysis of KEGG in the experimental group (abscissa is the name, ordinate is the value of relative abundance log2, different colors represent different groups. The different species with abundance and 0 in at least one group were not shown).

Effects of sevoflurane inhalation anesthesia on the expression of genes and metabolic pathways in the intestinal microbiome of mice: The differentially expressed genes of the experimental group on the 1st, 3rd, 7th and 14th days after anesthesia were identified. According to the KO classification of the KEGG library, only five types of genes, such as k00754 and k00680, were identified on the 1st day. In contrast, on the 3rd day, a large number of genes, such as k0184 and k02030, were found; on the 7th day, only 22 types of genes, such as k01992 and k01447, were identified, and on the 14th day, significant enrichment of k03088 and k07495 was detected. The numbers of differentially expressed genes on the 3rd and 14th days after sevoflurane inhalation anesthesia were significantly higher than those on the 1st and 7th days after anesthesia (**Figure 5**).

**Figure 6:**
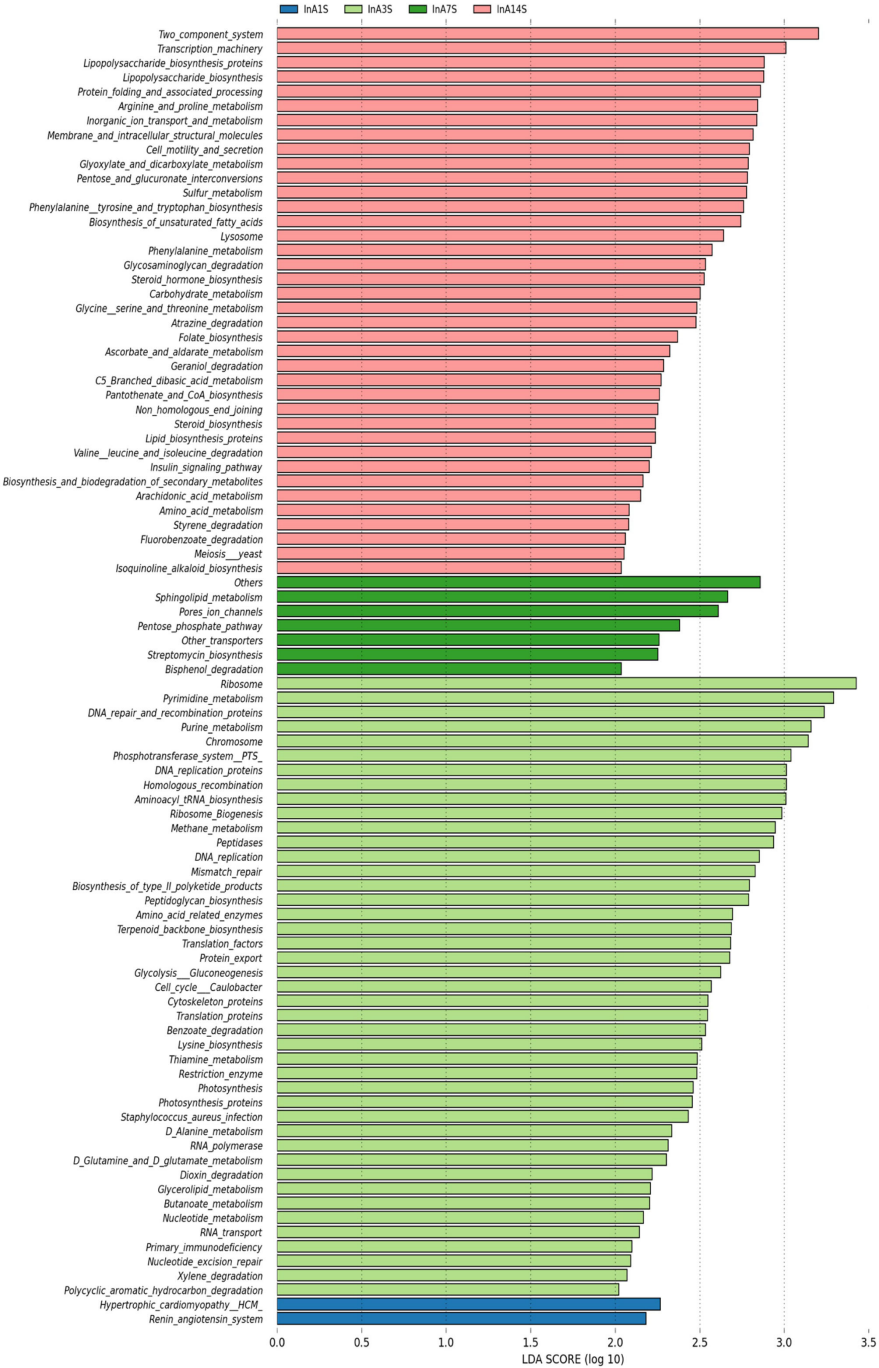
Differences in intestinal microbiome metabolic pathways. LEfSe analysis of experimental group KEGG L3 level difference pathway analysis. Different colors represent different samples at different time points in different experimental groups. The longer the color block length, the greater the impact of path on time.

On the 1st day, various metabolic pathways, such as ribosome, pyrimidine metabolism, DNA repair and replication proteins, purine metabolism, and chromosomes, were increased in the intestinal microbiome of the experimental group. On the 7th day, sphingolipid metabolism, porous ion channels, and the pentose phosphate pathway, among other pathways, were significantly increased. On the 14th day after anesthesia, two-component systems, transcription machinery lipopolysaccharide biosynthesis, protein folding and associated processing, arginine and proline metabolism and other metabolic pathways, were significantly enhanced (**Figure 6**).

**Figure 7:**
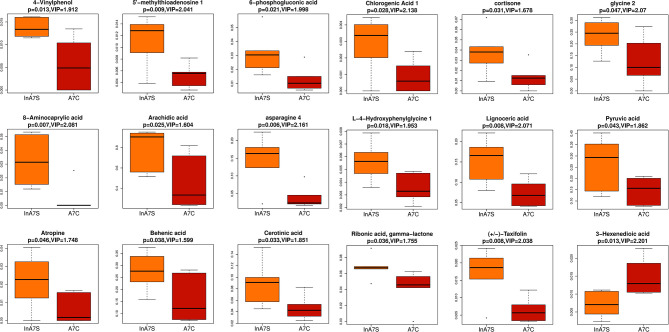
Boxplot was used to display the results of different metabolites. The difference of fecal metabolites between the experimental group and the control group on the 7th day was compared 4-Vinylphenol 、5-methylthioadenosine、6-phosphogluconic acid、8-Aminocaprylic acid、Arachidic acid、Asparagine 4 、Asparagine 4 、Behenic acid、Cerotinic acid、Chlorogenic Acid 1、Cortisone、glycine 2、L-4-Hydroxyphenylglycine、Lignoceric acid、Pyruvic acid、Ribonic acid, gamma-lactone、Taxifolin、3-Hexenedioic acid.

**Figure 8:**
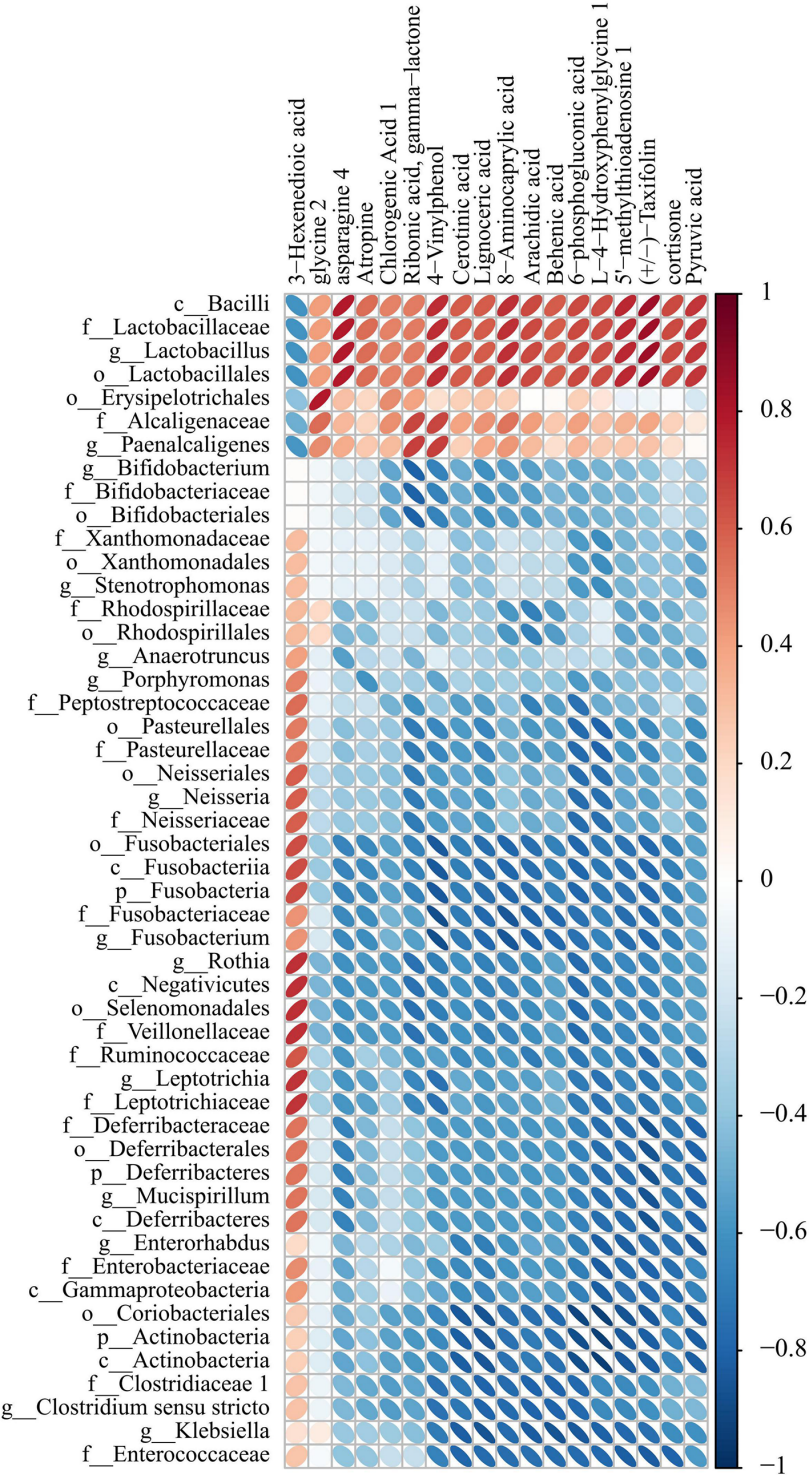
Correlation heat map analysis found the correlation between specific metabolites and microbial microbiome (P < 0.05). (below is the difference metabolite, on the right is the difference species, the color depth indicates the correlation size, blue indicates the negative correlation, red indicates the positive correlation).

We detected the intestinal metabolites on the 7th day after anesthesia, which were significantly different from those on the 1st, 3rd and 14th day: compared with the control group, the experimental group had more increased metabolites (**Figure 7**). Then, we analyzed the correlation between intestinal microbiome and metabolites, and divided the metabolites into two groups, which were positively correlated with intestinal microbiome: Asparagine 4, 8-Aminocaprylic acid、Lignoceric acid, Taxifolin, 5-methylthioadenosine 4-Vinylphenol, L-4-Hydroxyphenylglycine, 6-phosphogluconic acid, Arachidic acid、Chlorogenic Acid 1, Cortisone、Cerotinic acid, Ribonic acid, gamma-lactone, Behenic acid, Pyruvic acid, Atropine, Glycine 2. The metabolites with negative correlation were 3-Hexenedioic acid (**Figure 8**).

## Discussion

Whether sevoflurane inhalation anesthesia can induce changes in the intestinal microbiome is unclear. Our results show that the intestinal microbiome of mice is significantly altered after sevoflurane inhalation anesthesia. The diversity of the intestinal microbiome of the mice in the experimental group was significantly different from that of the mice in the control group on the 1st, 3rd, 7th and 14th days. The results obtained for the experimental group on the 1st, 3rd and 14th days were significantly different from those on the 7th day. The diversity of the intestinal microbiome was significantly decreased on the 7th day, which indicated that the effect of sevoflurane inhalation anesthesia on the intestinal microbiome might reach a peak on the 7th day after anesthesia, and this difference was decreased on the 14th day.

The previous results on the effect of isoflurane inhalation anesthesia on the intestinal microbiome differ from those obtained in our experiment. 1) In a previous study on the relationship between long-term neurocognitive impairment and the exposure of newborns to isoflurane, the authors anesthetized newborn mice and then tested their gut microbiome on the 35th day after anesthesia (Wang et al., 2019) with the aim of verifying the long-term effect of isoflurane inhalation anesthesia on the intestinal microbiome of newborn mice. 2) To determine whether intestinal dysbacteriosis mediates memory loss in elderly mice after anesthesia/surgery, a group of researchers not only gave isoflurane inhalation anesthesia to old mice but also performed surgery and administered local anesthesia and analgesia, and experimental samples were collected 48 hours after the operation to analyze the short-term changes in the intestinal microbiome (Jiang et al., 2019). 3) Another experiment on isoflurane inhalation anesthesia tested 20-22-week-old male and female mice, which are similar to old (24-week-old) mice. During aging, the physiological indexes of the body decline, the disease susceptibility increases, and the tolerance to external stimulation weakens and the intestinal microbiome also varies with age (Barreto et al., 2020). Moreover, the data from male and female mice show differences, and some studies have shown that female mice are prone to stress and that exposure to stress can lead to differences in the intestinal microbiome (Tsilimigras et al., 2018).

The advantage of our study was that the dynamic changes in the intestinal microbiome were closely monitored for a longer period of time. In addition, the inclusion of a negative control group eliminated the impact of environmental factors on the intestinal microbiome of mice. Moreover, all of the above-mentioned experiments used isoflurane as the anesthetic, and sevoflurane was not verified. In recent years, the frequency of the clinical application of isoflurane has decreased due to the pharmacological and side effects of this anesthetic, and sevoflurane inhalation anesthesia is being increasingly used. In our experiment, we studied the effect of sevoflurane inhalation anesthesia on the intestinal microbiome of adult male mice. We sequenced the 16S rRNA gene of fecal samples obtained on the 1st, 3rd, 7th and 14th days after sevoflurane inhalation anesthesia. We not only focused on the principle of a single variable but also set up a blank control group without anesthesia for a horizontal comparison to ensure the accuracy of the results. The results showed that the intestinal microbiome of the mice in the experimental group exhibited dynamic changes after sevoflurane anesthesia.

The effect of sevoflurane on the intestinal microbiome of mice may be through direct and indirect ways. Direct effects: In vitro experiments have shown that sevoflurane exert antibacterial effects on gram- and gram-positive bacteria and even multidrug-resistant bacteria. The numbers of bacteria associated with ventilator-associated pneumonia after coculture with sevoflurane (Serrano et al., 2017). Indirect effects: The indirect effect of sevoflurane on the intestinal microbiome might be realized through the brain-gut-bacteria axis (Gracie et al., 2019). A large number of studies have confirmed an interaction between the central nervous system and the gastrointestinal tract, which plays an important role in maintaining the stability of the internal environment of the digestive tract. Because gut microbes play a very important role in the brain-gut axis, we can regard the central nervous system, autonomic nervous system, gut nervous system, digestive tract and a wide variety of gut microbiomes as a whole, namely, as the brain-gut-bacteria axis (Bauer et al., 2019; Patterson et al., 2019). This function can be considered dual: down-up signal transduction from afferent fibers to the central nervous system and top-down signal transduction from efferent fibers projecting to the smooth muscle cells of the intestinal wall. This two-way interaction consists of down-top and top-down systems (Coss-Adame and Rao, 2014; Breit et al., 2018).

At present, the mechanism of action of sevoflurane on the central nervous system remains unclear. We speculated that sevoflurane affects the central system, and this effect can be transmitted to the digestive tract through the brain-gut axis to induce changes in the microbiome. In addition, the central nervous system receives signals from the lower end of the intestinal microbiome. This type of regulation is similar to a circular feedback regulation system. Although anesthetic drugs can be rapidly discharged from the body by inhalation, their effect on the intestinal microbiome resembles a “trigger switch,” which continues until balance in the body is achieved. On the 7th day after anesthesia, the results of metabonomics showed that there were a variety of metabolites increased in the experimental group. In addition to the basic metabolites related to body function, a specific metabolite 5-methylthioadenosine(MTA) was also found (**Figure 7**). The existing data show that MTA has a correlation with brain nerve function, which may play a positive role in the recovery of the disease. MTA can be used in the treatment of multiple sclerosis and other neurological diseases, and can be more safely and effectively reversed by oral route (Pramod et al., 2019). Methylthioadenosine phosphorylase (MTAP), which is related to its metabolism, is often absent in various cancers (Weder et al., 2020). Low expression of MTAP can predict the response to treatment, but the survival time is reduced (Jing et al., 2020). MTAP deficient glioblastoma cells showed high levels of DNA damage (Du et al., 2019). MTAP deficiency can also promote the formation of glioma stem cells (GSC), increase the expression of CD133 and enhance the tumorigenicity of glioblastoma cells, which is related to poor prognosis of patients (Hansen et al., 2019).

However, 3-hexenedioic acid, another specific metabolite, had the opposite effect with other metabolites in the results, and it had a negative correlation with the dominant microbiota on the 7th day after sevoflurane anesthesia. However, evidence suggests that glucose can be converted into 3-hexenedioic acid (Karlsson et al., 2018) by microorganisms. This may indicate that this substance may be the characteristic substance of bacterial differential expression. However, the above-described content is only hypothetical, and the specific mechanism needs to be further verified by future experiments.

Sevoflurane inhalation anesthesia increased the abundances of Bacteroides, Alloprevotella, and Akkermansia and decreased the abundances of Lactobacillus in the intestinal microbiome of mice, and this change lasted for a long time (14 days) or continuously (Wang et al., 2019).

A study of 116 fecal samples from 32 patients with pancreatic surgery showed that a decrease in Bacteroides might lead to an increased incidence of complications, such as infection and postoperative pancreatic fistula, after pancreatic surgery (Schmitt et al., 2019). A Japanese study conducted in 2018 also found that Bacteroides might exert a protective effect on the body and that the relative abundance of Bacteroides was significantly reduced in the stool of patients with coronary artery disease (CAD). The administration of live Bacteroides by gavage can decrease plaque inflammation, reduce the formation of atherosclerotic lesions, improve endotoxemia, reduce the production of intestinal microbial lipopolysaccharides, and effectively inhibit the proinflammatory immune response (Yoshida et al., 2018).

Akkermansia has been shown to exert anti-inflammatory effects (Earley et al., 2019; Huck et al., 2020) and enhance the integrity of intestinal epithelial cells (Reunanen et al., 2015). However, a recent study suggested that an increase in Akkermansia after pancreatic surgery might lead to increased complications, such as pulmonary embolism, infection, gastric emptying, and postoperative pancreatic fistula, in patients (Schmitt et al., 2019). The results of an experimental study on the changes in the intestinal microbiome of patients with gastric cancer during the perioperative period also showed that the abundances of Akkermansia were significantly increased after surgery compared with the levels prior to surgery and that the surgery decreased the levels of SCFAs that maintain the integrity of the intestinal mucosal barrier (Liang et al., 2019). Therefore, the advantages and disadvantages of Akkermansia to diseases remain to be discussed.

At present, few studies have investigated Alloprevotella, but most of the detected findings were related to an increased occurrence of diseases. A sequencing of 338 cases of colorectal cancer showed that high-risk tumors exhibit high expression of the IL23A and IL1RN genes and are rich in bacteria such as Alloprevotella (Ge et al., 2020). An experiment conducted in Japan confirmed that the bacterial diversity of oral cancer patients was increased and that Alloprevotella was more abundant than the other detected bacteria (Takahashi et al., 2019).

Therefore, the changes in the intestinal microbiome in mice after sevoflurane inhalation anesthesia result in increases and decreases of different bacteria. The current research on these bacteria shows that their changes will have an impact on the body, but whether this effect is protective or damaging to the body remains to be further proven and discussed.

In addition, the changes in the intestinal microbiome diversity are also accompanied by changes in bacterial differential gene expression and metabolic pathways. According to a query of the KEGG database, the changes in differential genes and pathways are related to the bacterial stress response (Herrou et al., 2017), glycolysis-related energy metabolism (Davies et al., 2011), the formation of galactose (Gao et al., 2019), and the target of antibiotic sterilization on bacteria (Liu et al., 2009; Liu et al., 2011; Sah et al., 2018) and the production of bacterial resistance (Behmard et al., 2019). However, at present, we can only observe the changes in genes and metabolic pathways. The specific increases or decreases in metabolites and the impact of these metabolites on the body need to be further verified by metabonomics and macro gene sequencing technology.

Because we didn’t detect blood gas analysis in the experiment, so the limitation of this study is that sevoflurane inhalation anesthesia might lead to changes in physiological indexes, such as hypoxia, hypothermia, hypercapnia and hypotension, in mice. Although these changes might be transient, they could also affect the composition of the intestinal microbiome. Wei Zhang et al. placed two groups of mice at high and low altitude for 30 days, and their results showed that the intestinal microbiome of HC (11% Oxygen) mice exhibited lower abundances of epsilon Proteobacteria and Actinobacteria compared with the intestinal microbiome of NC (16% Oxygen) mice (Zhang et al., 2018). Watkins C et al. collected 10 term infants with hypoxic-ischemic encephalopathy (HIE) and treated them with 33.5c total body cooling (TBC) for 72 hours. The control group consisted of nine healthy term infants. Fecal samples from both groups of 2-year-old infants were collected, and the results showed no significant effect on the intestinal microbiome in HIE/TBC infants compared with healthy infants (Watkins et al., 2019). Tripathi et al. simulated sleep apnea syndrome by exposing mice to intermittent hyperoxia and hypercapnia (IHH) for 6 weeks and found that this intervention could lead to changes in the intestinal microbiome and metabolites in mice (Tripathi et al., 2018). At present, few studies have investigated these contents, and the described interventions of mice that exhibit positive results are long term (30 days or 6 weeks). The short-term (4 hours) effects of hypoxia, hypothermia, and hypercapnia on the intestinal microbiome have not been verified, and the experimental results of a low body temperature 42 are negative. The intestinal microbiome changes caused by different blood pressures have not been verified. Therefore, whether the changes in physiological indexes caused by anesthesia over a short period of time have a significant impact on the intestinal microbiome need to be further explored, and their study might be very meaningful. Another limitation is that we only selected male mice for our experiment and did not analyze the influence of different genders on the effects of sevoflurane inhalation anesthesia on the intestinal microbiome. In the future, we will perform relevant experiments to verify our findings.

## Conclusions

Sevoflurane inhalation anesthesia is associated with changes in the internal microbiome and metabolites. This change may be completed through the brain gut axis, while sevoflurane inhalation anesthesia may change the intestinal microbiome for as long as 14 days or longer.

## Data Availability Statement

The data presented in the study are deposited in the NCBI(https://www.ncbi.nlm.nih.gov/) repository, accession number is PRJNA688133.

## Ethics Statement

Experiments were performed under a project license (no.: KY2018-02) granted by regional ethics board of Harbin Medical University Cancer Hospital on December 2018, in compliance with Chinese Guidelines for the Care and Use of Animals. The chairperson of the ethics committee is Changhong Zhao.

## Author Contributions

All authors participated in the design, interpretation of the studies and analysis of the data and review of the manuscript. KY, CW, and CH designed the research and wrote the manuscript. ZZ, NG, and XL implemented the experiments and performed the data analysis. MY, YP, and XM reviewed and edited the manuscript. All authors contributed to the article and approved the submitted version.

## Funding

Supported by the National Natural Science Foundation of China(Nos. 81571871,81770276,U20A20366,81704165), Nn10 program of Harbin Medical University Cancer Hospital, Natural Science Foundation of Heilongjiang Province (YQ2020H038).

## Conflict of Interest

The authors declare that the research was conducted in the absence of any commercial or financial relationships that could be construed as a potential conflict of interest.
